# Three Cases of Severe Placental Abruption as a First Symptom of Preeclampsia

**DOI:** 10.1155/2021/3863607

**Published:** 2021-07-23

**Authors:** Anastasia Mikuscheva, Finn Strassding, Elliot MacKenzie

**Affiliations:** ^1^Zollikerberg Hospital, Zollikerberg, Switzerland; ^2^Department of Gynecology and Obstetrics Dunedin Public Hospital, New Zealand

## Abstract

Placental abruption is often referred to in the literature as a complication of preeclampsia. We report 3 recent cases where the first symptom of preeclampsia was placental abruption. All women were previously healthy and in their first ongoing pregnancy. All had been seen by obstetricians for regular pregnancy checkups. None of the patients had a preexisting diagnosis of preeclampsia. Only one of the patients had risk factors for preeclampsia and occasional hypertension. In all cases, laboratory signs of preeclampsia were abnormal intra- or immediately postpartum. One fetus died in utero, and the other two pregnancies fortunately showed a favourable outcome.

## 1. Introduction

Most preeclampsia courses are mild and resolve in complete restitution in the postpartum period. Unfortunately, it is currently not possible to reliably identify those patients with few or no risk factors who will have fulminating courses and need more close observation as in- or outpatients. Apart from the placenta, there is no monocausal etiology of preeclampsia. Genetic predisposition, preexisting medical conditions, and fetal growth modulate the maternal systemic response to the placenta. The development of classical preeclampsia with its cardinal symptom hypertension and proteinuria is currently regarded as a 2-stage process. The first stage includes defects of early placentation like poor trophoblast uterine invasion, impaired transformation of the uterine spiral arteries to high capacity and low impedance vessels, and/or abnormalities in the development of chorionic villi [1]. Impaired spiral artery remodelling seems essential for this process and leads to placental hypoperfusion and oxidative stress. The second phase consists of an exaggerated maternal immune reaction to these processes [1]. Lately, however, concerns have been raised in the literature that impaired placentation does not explain late-onset preeclampsia as placentae of these patients are often normal sized and the affected pregnancies are only complicated by IUGR in 15% of cases. Especially when placental disease occurs near-term, consideration should be given to the possibility that the placenta is a perfusion-dependent organ and that in these cases, impaired cardiovascular function of the mother may cause placental dysfunction, rather than faulty placentation [2].

High flow resistance in the uterine arteries and in the umbilical artery in the 2nd and 3rd trimesters are ultrasonographic correlates and or risk factors for impaired placental structure and function and come with an increased risk for obstetric complications [3].

Malplacentation seems to be more frequently associated with early-onset preeclampsia which can lead to fetal growth restriction. On the other hand, the role of malplacentation in late-onset preeclampsia is not so clear. It rather seems to be caused by an impaired maternal cardiovascular response to pregnancy [4]. The concept of ischemic placental disease, a unifying classification for preeclampsia, IUGR, and abruption, is well supported in the literature. Clinical studies of Doppler findings of both uterine and umbilical arteries, as well as placental histologic findings, confirm the theory that malplacentation is a cause of ischemic placental disease. Even though it is plausible that an ischemic placenta is the result of faulty placentation, it is a syndrome rather than one disease with a spectrum of symptoms that can occur preceding or following each other at variable time points. It has been postulated that the development of preeclampsia and IUGR depends on the extent or failure of trophoblast invasion. In our series, none of the pregnancies were complicated by IUGR and the preeclampsia occurred concurrently or postpartum.

Placental abruption is defined as a premature separation of the placenta from the uterine wall before birth of the fetus. The criteria needed to define placental abruption as “severe” should be clinically meaningful and should include at least 1 maternal (disseminated intravascular coagulation, hypovolemic shock, blood transfusion, hysterectomy, renal failure, or inhospital death), fetal (nonreassuring fetal status, intrauterine growth restriction, or fetal death), or neonatal (neonatal death, preterm delivery, or small for gestational age) complications. The prevalence of placental abruption ranges from 0.3 to 1% [5]. Risk factors for abruption include maternal hypertensive disorders, advanced maternal age, multiparity, smoking, cocaine use, previous caesarean delivery, uterine surgery, and short interpregnancy interval [6]. Clinical findings include vaginal bleeding, abdominal pain, tetanic uterine contractions, and an abnormal CTG [7].

Here, we report 3 recent cases of a first manifestation of preeclampsia through severe placental abruption.

## 2. Case Report

A 33-year-old patient 1G0P at 35 + 2 gestation presented to our obstetric unit because of severe abdominal pain that had started a few hours before presentation. The patient had no significant medical or obstetric history, and the course of this pregnancy had been uneventful. She had last seen her gynaecologist 4 weeks prior to this presentation because of a self-limited episode of generalized edema, however because hypertension and proteinuria had been excluded was discharged home without further follow-up. On presentation, no fetal heart beat could be found on CTG monitoring which was confirmed on an ultrasound scan. The scan also showed a retroplacental hematoma of approximately 13 × 8 cm. On clinical exam, the abdomen was hard and tender. The patient was normocardic and normotensive, however was extremely pale; and pale lips were especially noted.

Laboratory examinations showed an Hb 116 g/l, platelets of 211 G/l, a haptoglobin of <0.01, an ASAT 40 U/L, an LDH 312 U/L, and a creatinine 86 *μ*mol/L as well as a uric acid of 357 *μ*mol/L.

The digital vaginal exam showed an unfavourable cervix, and there was no vaginal bleeding. A delivery by emergency caesarean section was indicated. Intraoperatively approximately 1 L of coagula was emptied. The uterus was livid and showed signs of intramural bleeding compatible with a beginning Couvelaire-uterus. The lividity improved intraoperatively; hence, the uterus was left in situ.

On the first postoperative day, the patient had a large subcutaneous bleed and hematoma formation on the left side of her caesarean scar which required operative evacuation. She was admitted to our intensive care unit and needed 3 units of packed red blood cells with an Hb of 58 g/l. She developed massive generalized edema, headaches, visual changes, and hypertension up to 150/105 mmHg. The platelets dropped to 88 G/L. The blood pressure and laboratory findings improved postoperatively without further therapy. She was discharged home in good physical condition on postoperative day 7 with a recommendation for antiphospholipid syndrome testing 6 weeks postpartum.

The 2nd patient was a 34-year-old 2G0P. She presented at 38 + 4 weeks gestation with heavy vaginal bleeding. Her obstetric history included a missed abortion 1 year prior. In this pregnancy, bilateral notching in the uterine arteries was noted on anatomy scan at 21 weeks gestation which was performed at a private ultrasound practice. She was put on Aspirin 150 mg, however advised that the recommended time frame for initiation of Aspirin therapy had been missed. She regularly measured her blood pressure at home and noted occasional high blood pressures up to 140/90; however, these episodes were self-limited and never reached levels requiring antihypertensive therapy. She had been checked by her gynaecologist regularly, and no proteinuria was seen.

On presentation, a fetal bradycardia was noted on ultrasound scan, placental abruption was suspected, and an emergency caesarean section performed. The patient was delivered of a male fetus with an APGAR 3/7/7, NsvpH 6.92, and NsapH 6.98. The neonate was initially nonresponsive to stimulation, asystolic, and apnoeic. After 30 seconds of reanimation, cardiac activity returned. The neonatal Hb was stable postoperatively, and no blood transfusions were necessary.

On the 4th postoperative day, the patient had hypertension up to 155/90 mmHg. She complained of visual changes, edema, and a mild headache. Laboratory findings included elevated liver enzymes (ASAT 81 U/l and ALAT 64 U/l) and a borderline protein/creatinine ratio (24.5 mg/mmol). Based on the hypertension and elevated liver enzymes, we diagnosed preeclampsia. During the hospitalization period, the laboratory findings improved and the patient could be discharged home in good physical condition. The neonate had to be transferred to the neonatology department of the university children's hospital for further investigations; however, his cardiovascular condition improved within a few days, and he could be transferred back to our neonatology unit.

The third patient was a 34-year-old 1G0P at 38 + 2 weeks gestation. She presented to our obstetric unit with vaginal bleeding. Prior to presentation, the pregnancy had been completely uneventful. The CTG showed a nonreassuring fetal state, and emergency caesarean section was performed. Based on the experiences described above, an intraoperative blood and urine sample were sent for preeclampsia investigations. Laboratory findings showed a protein-creatinine ratio of (156.6 mg/mmol). The blood pressure rose to 144/91 on the first postoperative day. The male fetus was delivered with an APGAR of 3/7/8 and needed admission to the neonatal intensive care unit and CPAP ventilation. He recovered well, and the patient and neonate could be discharged home in good physical condition on postoperative day 5.

## 3. Discussion

Placental abruption is a known and potentially life-threatening complication of hypertensive and preeclamptic disease for mother and fetus. Here, we report 3 cases were placental abruption occurred as a first manifestation of preeclamptic disease. Even though it seems plausible that ischemic placental disease is the result of faulty placentation, it is a syndrome rather than one disease and hence consists of a spectrum of symptoms that can occur preceding or following each other at variable time points. It has been postulated that the development of preeclampsia and IUGR depends on the extent (or failure) of trophoblast invasion. In our series, none of the pregnancies were complicated by IUGR and preeclampsia occurred concurrently with abruption or postpartum. Hence, we think that preeclampsia or ischemic placental disease cannot simply be described as a two-stage process with faulty placentation as the sole underlying cause. It rather seems to be a syndrome with different etiologies that unite in a common pathway.

Hypertension in preeclampsia is often described as a maternal compensatory mechanism for the blood flow deficiency in the fetus. The mother supposedly develops hypertension to increase blood flow towards the fetus, usually at the end of the second or third trimester of pregnancy [8]. As logical as this concept seems, it is contradicted by the fact that in a substantial amount of cases, hypertension occurs intra- or postpartum and that tight control of maternal blood pressure with antihypertensives does not lead to IUGR as shown in the CHIPS trial [9].

We do believe that impaired placentation in early pregnancy was responsible for abruption at least in the 2nd case. This assumption is backed up by the notching in the uterine arteries on anatomy scan as well as occasional hypertension in the mother prior to delivery. We postulate that testing of the sFLT1/PlGF ratio would have been indicated in this pregnancy and might have led to an earlier suspicion for placental disease before it led to abruption.

The first described pregnancy, however, was uneventful apart from generalized edema that occurred 1 month prior to presentation and terminated in the most tragic manner. It is arguable whether the generalized edema the patient presented to her gynaecologist with should have prompted sFLT/PlGF testing. Preeclampsia has been described as an endothelial disease where malperfusion of the placental tissue leads to tissue necrosis and release of reactive oxygen species that cause endothelial dysfunction and capillary leak [8]. We hypothesize that it is hence not unreasonable to test placental function in a patient presenting with edema only even when hypertension and or proteinuria is absent at the time of presentation.

The third patient again showed no risk factors for placental disease. Unfortunately, none of the placentas of the described cases were sent for pathologic investigations.

On discharge, we recommended close monitoring of future pregnancies to all three patients including uterine artery Dopplers, Aspirin from the first trimester, and timely and regular sFLT/PLGF testing at the occurrence of even small symptoms.

## 4. Conclusion

Preeclampsia is one of the most common causes for placental abruption. Both preeclampsia and abruption are different manifestations of ischemic placental disease the etiology of which is to date incompletely understood. Current concepts fail to provide plausible explanations to the broad spectrum of symptoms that this syndrome can manifest with. Our case series demonstrates that preeclampsia does not necessarily have to precede abruption but can occur simultaneously or afterwards and always has to be considered as a differential diagnosis or cause and tested for when abruption occurs.
